# Recombinant expression of receptor binding domains of all eight subtypes of botulinum neurotoxin type A for generation of antitoxins with broad reactivity

**DOI:** 10.12688/f1000research.160607.1

**Published:** 2025-02-05

**Authors:** Nga Quynh Pham, Tam Trang Mai, Tran Bao Anh Dang, Ly Huong Tran, Quynh Mai Vu, Chien Trong Nguyen, Anh Thi Phuong Tran, Tran Nhat Minh Dang, Van Anh Tran, Thinh Huy Tran, Van Khanh Tran, Hoa Quang Le

**Affiliations:** 1 School of Chemistry and Life Sciences, Hanoi University of Science and Technology, Hanoi, Vietnam; 2 High School for the Gifted in Natural Sciences, Vietnam National University, Hanoi, 120558, Vietnam; 3 Hanoi Medical University, Hanoi, 11521, Vietnam; 4 Center for Gene and Protein Research, Hanoi Medical University, Hanoi, 11521, Vietnam

**Keywords:** botulinum neurotoxins, botulism, receptor-binding domain, HC, recombinant proteins, neutralization, VHH, nanobody, antitoxin

## Abstract

**Background:**

Botulinum neurotoxin type A (BoNT/A) represents a major threat to global public health because of its most potent toxicity with the longest persistence. Several camelid single-domain antibodies (or VHHs) have been reported to exhibit high neutralizing activity against the receptor binding domain (H _C_) of the BoNT/A subtype used to generate them. However, it remains unclear if these VHHs can neutralize effectively H _C_ of other BoNT/A subtypes. This study aimed to generate H _C_ domains of all eight BoNT/A subtypes and to screen for VHHs with broad reactivity against these domains.

**Methods:**

H _C_ domains of BoNT/A1-A8 were recombinantly produced in *Escherichia coli.* The *bont/H _C_A1* fragment was amplified from sludge sample and cloned into pET45b vector by Gibson assembly. Expression vectors for H _C_ domains of BoNT/A2-A8 were derived from pET45b-H _C_A1 by site-directed mutagenesis and/or in-house gene synthesis. Similarly, VHHs were synthesized and cloned into pET22b vector. Recombinant protein were purified by Ni-NTA spin columns and analyzed by SDS-PAGE. ELISA was used to confirm the antigenicity of H _C_ domains and to evaluate the reactivity of VHHs to these domains.

**Results:**

SDS-PAGE analysis and ELISA results with commercial polyclonal antibody demonstrated the H _C_ domains of all eight BoNT/A subtypes were correctly produced. ELISA results using a VHH panel indicated that, apart from ciA-C2, a well-characterized VHH specific for H _C_ of BoNT/A1, two new VHHs were found to recognize the H _C_ domains of all BoNT/A subtypes, of which VHH-A3 displayed EC _50_ values for these domains close to those of ciA-C2.

**Conclusion:**

This study provided a resource to comprehensively identify antitoxins conferring broad protection against BoNT/A.

## Introduction

Botulinum neurotoxins (BoNTs) are the most toxic substances known to the humankind with lethal dose values in the range of nanogram per kilogram body weight scale. ^1^ Most commonly produced by *Clostridium botulinum*, these toxins are proteins composed of a 50-kDa light chain (LC) linked to a 100-kDa heavy chain (HC) via a disulfide bond. The LC fragment contains a zinc-protease specific domain, whereas the HC consists of an N-terminal translocation domain (H _N_) and a C-terminal receptor-binding domain (H _C_). ^2^ The mode of action of BoNTs includes three steps. In the first place, the H _C_ domain of BoNTs bind specifically to peripheral nerve terminals via polysialoganglioside and synaptic vesicle receptors. Subsequently, BoNTs enter into nerve terminals by endocytosis. Under acidic conditions, the H _N_ domain translocates the LC into the nerve terminal cytosol where the latter cleaves one of three soluble N-ethylmaleimide-sensitive factor attachment protein receptors (SNARE) that are involved in neurotransmitter release, thereby causing nerve paralysis. ^2^

BoNTs are traditionally classified into seven serotypes (BoNT/A–BoNT/G), of which BoNT/A represents a great threat to humans because of its most potent toxicity with the longest duration of paralysis. ^3^ In addition, BoNT/A is categorized into eight subtypes (BoNT/A1–A8) with significant levels of protein sequence differences (up to 12.3%), ^4^ which complicates the development of a broadly protective monoclonal antitoxin.

Botulinum intoxication is fatal in 5–10% of cases and requires early treatment with antitoxin. Currently, the only available antitoxins for botulism are the heptavalent botulinum antitoxin (HBAT), which contains fragments of immunoglobulins from horses vaccinated with all seven traditionnal serotypes of BoNTs, and BabyBIG, which consists of polyclonal antibodies from human immunized with recombinant botulinum vaccine for serotypes A and B. However, these antitoxin types have limitations due to adverse side effects, limited availability and exorbitant cost. ^5^ To overcome these drawbacks, neutralizing monoclonal antibodies (mAbs) against BoNT/A, B, E, and F, which cause human botulism, have been generated. ^6– 9^ It has been shown that a combination of several mAbs is required to efficiently neutralize subtypes belonging to a BoNT serotype. ^8, 9^ Another strategy to combat botulism is to develop camelid single-domain antibodies (sdAbs), also referred to as VHHs or nanobodies, that can neutralize BoNTs via the interactions with the functional domains of the toxins. Several VHHs with high affinity against H _C_ domain of a BoNT/A subtype have been shown to display protective activity when challenged with the same toxin in animal models. ^10– 12^ However, it remains unclear if these VHHs can neutralize effectively other subtypes of BoNT/A. This is because of the lack of a comprehensive toxin resource available for all subtypes of BoNT/A. Here, for the first time, the generation of recombinant H _C_ domains of BoNT/A1-A8 was described. These proteins were then used to characterize a panel of VHHs targeting BoNT/A1 with unknown binding sites in order to identify novel VHHs with broad reactivity against all subtypes of BoNT/A.

## Methods

### Materials

*Clostridium botulinum* genomic DNA carrying the *bont/A1* gene was extracted from an enrichment of a sludge sample from Hanoi, Vietnam.

Oligonucleotides were synthesized by Macrogen (Korea). Expression vectors used in this study comprised pET-45b and pET-22b (Novagen, cat. number 71327-3 and 69744-3, respectively). Hosts used for recombinant protein production were *E. coli* BL21(DE3) and *E. coli* Rosetta™ 2(DE3) (Novagen, cat. number 69450 and 71397, respectively).

All other reagents were from Thermo Scientific™, New England Biolabs, Merck, Qiagen, Tetracore, Immunology Consultants Laboratory, Vazyme, and Himedia unless otherwise stated.

### Construction of expression vectors for BoNT/A1-A8 production

The *bont/H _C_A1* fragment (residues 871-1296 of BoNT/A1) was amplified from extracted *C. botulinum* DNA and cloned into pET45b vector with an N-terminal His-tag by NEBuilder ^®^ HiFi DNA Assembly Master Mix (New England Biolabs, cat. number E2621L). ^13^ Expression vectors for H _C_ domains of BoNT/A2-A8 ( Table 1) were derived from pET45b-H _C_A1 by site-directed mutagenesis and/or in-house gene synthesis. ^14– 20^ All vectors were sent for sequencing to verify the accuracy of the constructs. Sequencing results can be found in the Sequence Read Archive under accession number PRJNA1206782.

**Table 1.** Accession numbers of BoNT/A subtypes used in this study.

BoNT/A subtypes	Accession numbers
A2	WP_061323842.1
A3	WP_012301031.1
A4	WP_012720356.1
A5	WP_078992015.1
A6	ACW83608
A7	AFV13854
A8	AJA05787

### Construction of expression vectors for VHH production

Genes encoding VHHs (A1, A3, A16, A17, A18, and ciA-C2) from previously reported studies ^10, 21^ were codon-optimized for expression in *E. coli* and synthesized by Genscript. These genes were inserted into the pET22b expression vector which was modified to carry a FLAG tag (DYKDDDDK) at the C-terminal end for detection. ^22^ All vectors were sent for sequencing to verify the accuracy of the constructs. Sequencing results can be found in the Sequence Read Archive under accession number PRJNA1206786.

### Expression and purification of recombinant proteins

H _C_ domains of BoNT/A1-A8 and VHHs were produced in *E. coli* Rosetta 2(DE3) and *E. coli* BL21(DE3), respectively. Bacteria carrying expression vectors were cultured at 37°C in LB medium (Himedia, cat. number 81254) supplemented with appropriate selecting antibiotics until OD _600_ ~ 0.6 – 0.8 and then induced with 0.5 mM IPTG (Thermo Scientific, cat. number R0392) at 20°C for 12 hours. The His tagged recombinant proteins were purified by affinity chromatography using Ni-NTA spin columns (Qiagen, cat. number 31014) under native conditions according to the manufacturer’s instructions and then analyzed by SDS-PAGE.

### ELISA assays

The reactivity of VHHs and commercial Rabbit Anti-Botulinum Toxin A and B IgG (Tetracore, cat. number TC-7007-001) against purified recombinant H _C_ domains of BoNT/A1-A8 were tested by ELISA. High Bind Stripwell™ Microplates (Corning, cat. number 07-200-24) were coated with 2 μg/mL recombinant antigens in carbonate buffer (Thermo Scientific, cat. number CB01100) at 4°C overnight. Plates were then blocked by 1% bovine serum albumin (Sigma-Aldrich, cat. number A2058) for 2 h at 37°C. VHHs in serial dilutions and commercial polyclonal antibody were added to the wells and incubated for 2 h at 37°C. After washing, anti-DYKDDDDK (Flag) Antibody Rabbit - HRP Conjugated (for VHHs) and HRP Conjugated Goat anti-Rabbit IgG h+l Antibody (for polyclonal antibody) (Immunology Consultants Laboratory, cat. number RFLG-45P-Z and GGHL-15P, respectively) were added and incubated for 1 h at 37 °C. Plates were then washed six times with PBST (Sigma-Aldrich, cat. number P3563) and reactions were developed with TMB substrate (Abcam, cat. number AB171523) and read at 450 nm. EC _50_ values were calculated via non-linear regression analysis using GraphPad Prism.

## Results and discussion

**Figure 1.**
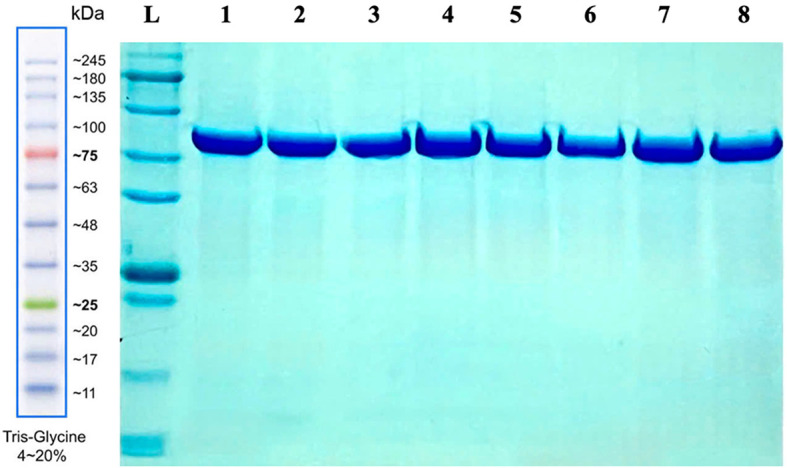
SDS-PAGE analysis of purified recombinant BoNT/A H _C_ domains.

Due to the extremely high toxicity of BoNTs, the development of antitoxins against them is of major interest for therapeutic applications. The target for antitoxin can be each of the three structural domains of BoNTs: (i) H _C_ responsible for receptor binding; (ii) H _N_ for toxin translocation; and (iii) LC for cleavage of SNARE proteins. Among these domains, the H _C_ fragment is the target of choice for the generation of antidote to BoNT intoxication, ^23^ as well as for the development of vaccines ^24^ and for intracellular delivery of cargo molecules specifically to neurons. ^25^ In the present study, we focused on the production of the H _C_ fragments of all subtypes of BoNT/A because of its extremely high toxicity, long persistence, and high sequence divergence among subtypes. However, only *bont/A1* gene was available in our group. Consequently, we opted for an approach that involved both site-directed mutagenesis from H _C_A1 construct and in-house gene synthesis to generate expression vectors for H _C_ domains of BoNT/A2-A8. ^14– 20^ These recombinant proteins were expressed in the *E. coli* Rosetta 2(DE3) and purified by nickel affinity chromatography. SDS-PAGE analysis showed that only one band was observed for all purified samples at the expected molecular mass of 50 kDa ( Figure 1), suggesting that H _C_ fragments of BoNT/A1-A8 were successfully expressed and prepared. The purified proteins were then evaluated by ELISA for antigenicity using the Rabbit Anti-Botulinum Toxin A and B IgG. All samples were recognized by the commercial polyclonal antibody, ^26^ which indicated that the receptor binding domains of all BoNT/A subtypes were correctly expressed in *E. coli* Rosetta 2(DE3).

To our knowledge, only three VHHs neutralizing BoNT/A by binding to the H _C_ domain have been described in the literature, ^10– 12^ of which ciA-C2 have been extensively characterized for the inhibition mechanism on the BoNT/A1. ^11^ In a separate report, 18 VHHs have been identified for their specific recognition of BoNT/A1 ^21^ but their binding sites were unknown. We therefore performed a systematic screening of a panel of five most promising VHHs from this report and ciA-C2, on the H _C_ fragments of BoNT/A1-A8 by ELISA. All the VHHs were successfully expressed and purified. ^22^ Screening results clearly indicated that apart from ciA-C2, two nanobodies (VHH-A1 and VHH-A3) could recognize the H _C_ domains of all BoNT/A subtypes with VHH-A3 exhibiting significantly higher affinity than VHH-A1. ^27^ Consequently, only ciA-C2 and VHH-A3 were characterized in subsequent experiments. According to the EC _50_ values calculated using ELISA ( Table 2), ^28^ both ciA-C2 and VHH-A3 exhibited the highest affinity for the H _C_A1 (EC _50_ = 11.0 and 24.0 nM respectively). This is not surprising, because these two VHHs were generated based on the selection with BoNT/A1. ^11, 21^ VHH-A3 displayed an intermediate affinity for H _C_A4 (EC _50_ = 46.0 nM) and comparably low affinity for the remaining subtypes. In comparison to ciA-C2, VHH-A3 displayed similar EC _50_ values for H _C_ domains of BoNT/A2, A3, A5, A6, A7, and A8. Concerning ciA-C2, the binding mechanism of this VHH to H _C_A1 involves a cation-π interaction and multiple hydrogen bonds between CDR1 and residues K289, N318 and D419 of H _C_A1. In addition, CDR2, CDR3, FR2, FR3, and FR4 of ciA-C2 also participate in the binding to H _C_A1 through hydrogen bonds with residues T193, H194, Y242, T276, E423 and a hydrophobic interaction with P425 of the domain. ^11^ Consistent with these structural observations, the affinity of ciA-C2 was least affected for H _C_A4 (EC _50_ = 26.2 nM) with only a T193 to P193 replacement, ^16^ whereas it was most affected for H _C_A2, H _C_A3 and H _C_A8 (EC _50_ ≥ 80.4 nM) containing three major substitutions T193P, H194R, and P425S. ^14, 15, 20^ These data underline the importance of the structural studies of VHHs in order to generate antitoxins with a broad protection to BoNTs. Furthermore, considering the sequence divergence among H _C_ domains of BoNTs ^29^ and most studies so far use BoNT/A1 as the selection agent to generate VHHs, it would be of interest to include a divergent H _C_ domain, for instance, H _C_A2, H _C_A3 or H _C_A8, during the selection steps in order to obtain VHHs having high affinity against these domains. Similarly, these recombinant fragments could be combined with H _C_A1 for the development of vaccines or polyclonal antitoxins with broad potency compared to conventional approach using only one BoNT/A subtype for immunization.

**Table 2.** EC _50_ values of ciA-C2 and VHH-A3 against H _C_ domains of BoNT/A1-A8.

BoNT/A subtypes	ciA-C2	VHH-A3
**A1**	11.0	24.0
**A2**	80.4	93.5
**A3**	83.8	88.9
**A4**	26.2	46.0
**A5**	51.4	67.6
**A6**	77.5	97.1
**A7**	62.3	73.3
**A8**	109.3	98.2

In summary, this study provided recombinant H _C_ domains of all BoNT/A subtypes, which could be used for the development of antitoxins and vaccines against BoNTs. This study also identified two new nanobodies, VHH-A1 and VHH-A3, capable of binding to all BoNT/A H _C_ domains. However, one question remains unsolved in this study, whether the VHH-A1, VHH-A3 and ciA-C2 would bind to a distinct, non-overlapping epitope. Further research is on-going to resolve this question and to improve neutralizing activity of ciA-C2 through the generation of heterodimers.

## Ethics and consent

Ethical approval and consent were not required.

## Data Availability

Figshare: Raw data of ELISA results for reactivity of H _C_A1-H _C_A8 to VHHs and Rabbit Anti-Botulinum Toxin A and B IgG; for determination of EC _50_ values of ciA-C2 and VHH-A3 against HCA1-HCA8. Doi: https://doi.org/10.6084/m9.figshare.28171994. ^30^ This project contains the following underlying data:
• ELISA raw data.xlsx Data are available under the terms of the Creative Commons Zero “No rights reserved” data waiver (CC0 1.0 Public domain dedication). Dataset from NCBI Sequence Read Archive: Sequencing results of H _C_-BoNT/A subtypes. Accession number PRJNA1206782; https://www.ncbi.nlm.nih.gov/sra/?term=PRJNA1206782. Dataset from NCBI Sequence Read Archive: Sequencing results of VHHs against H _C_-BoNT/A subtypes. Accession number PRJNA1206786; https://www.ncbi.nlm.nih.gov/sra/?term=PRJNA1206786. Figshare: Supplement I for “Recombinant expression of receptor binding domains of all eight subtypes of botulinum neurotoxin type A for generation of antitoxins with broad reactivity”. Figshare: Construction of pET45b-HCA1 plasmid. Doi: https://doi.org/10.6084/m9.figshare.28159514.v1. ^13^ This project contains the following extended data:
• Supplement I.pdf Figshare: Construction of pET45b-HCA2 plasmid. https://doi.org/10.6084/m9.figshare.28159544.v1. ^14^ This project contains the following extended data:
• Supplement II.pdf Figshare: Construction of pET45b-HCA3 plasmid. https://doi.org/10.6084/m9.figshare.28159553.v1. ^15^ This project contains the following extended data:
• Supplement III.pdf Figshare: Construction of pET45b-HCA4 plasmid. https://doi.org/10.6084/m9.figshare.28159559.v1. ^16^ This project contains the following extended data:
• Supplement IV.pdf Figshare: Construction of pET45b-HCA5 plasmid. https://doi.org/10.6084/m9.figshare.28159574.v1. ^17^ This project contains the following extended data:
• Supplement V.pdf Figshare: Construction of pET45b-HCA6 plasmid. https://doi.org/10.6084/m9.figshare.28159586.v1. ^18^ This project contains the following extended data:
• Supplement VI.pdf Figshare: Construction of pET45b-HCA7 plasmid. https://doi.org/10.6084/m9.figshare.28159592.v1. ^19^ This project contains the following extended data:
• Supplement VII.pdf Figshare: Construction of pET45b-HCA8 plasmid. https://doi.org/10.6084/m9.figshare.28159598.v1. ^20^ This project contains the following extended data:
• Supplement VIII.pdf Figshare: Construction of pET22b-VHH plasmids. https://doi.org/10.6084/m9.figshare.28159622.v1. ^22^ This project contains the following extended data:
• Supplement IX.pdf Figshare: Reactivity of HCA1-HCA8 to the Rabbit Anti-Botulinum Toxin A and B IgG. https://doi.org/10.6084/m9.figshare.28159754.v1. ^26^ This project contains the following extended data:
• Supplement X.pdf Figshare: Reactivity of HCA1-HCA8 to VHHs. https://doi.org/10.6084/m9.figshare.28159982.v1. ^27^ This project contains the following extended data:
• Supplement XI.pdf Figshare: Determination of EC _50_ values of ciA-C2 and VHH-A3 against HCA1-HCA8. https://doi.org/10.6084/m9.figshare.28160042.v1. ^28^ This project contains the following extended data:
• Supplement XII.pdf Figshare: The overall amino acid sequence identity among BoNT/A subtypes using, A1 as the benchmark. https://doi.org/10.6084/m9.figshare.28160051.v1 ^29^ This project contains the following extended data:
• Supplement XIII.pdf Data are available under the terms of the Creative Commons Zero “No rights reserved” data waiver (CC0 1.0 Public domain dedication). The funders had no role in study design, data collection and analysis, decision to publish, or preparation of the manuscript.
