# Supplementary material for: Recombinant expression of receptor binding domains of all eight subtypes of botulinum neurotoxin type A for generation of antitoxins with broad reactivity
Source: F1000Res. 2025 Feb 5;14:163. [Version 1] doi: 10.12688/f1000research.160607.1 (PMC11880751; doi:10.12688/f1000research.160607.1)
Supplement: Peer review history

# Reviewer report: Reviewer response for version 1

**DOI:** 10.5256/f1000research.176528.r366729

**Published:** 2025-03-04

**Authors:** Charles B Shoemaker

**Affiliations:** 1 Tufts University, Medford, Massachusetts, USA

In this manuscript, the author describe the creation of a set of Botulinum neurotoxin serotype type A (BoNT/A) heavy chain domain (AHc) representing each of the eight reported BoNT/A subtype natural variants. They use this resource to screen the AHc subtype specificity of several single-domain antibodies (sdAbs) that are reported to bind to AHc. Comprehensive methods and construction details are provided and the work appears well performed. 

 General comments:

- The authors suggest the primary accomplishment is their creation of “a resource to comprehensively identify antitoxins conferring broad protection against BoNT/A” which seems a substantial overstatement as the resource is simply a panel of expressed receptor-binding domain subunits of known amino acid sequence. Most capable labs could create a similar set by ordering and cloning a panel of synthetic coding DNA into an expression vector. Furthermore, identifying broad subtype antibody binding to AHc does not demonstrate their antitoxin potential as the VHH binding site must also be at a site that interferes with toxin binding which requires neutralization studies.
- Authors claim that the ciA-C2 specificity for AHc is unknown though information on subtype specificity was provided in the manuscript cited as the source of this information.

Is the work clearly and accurately presented and does it cite the current literature?

Yes

If applicable, is the statistical analysis and its interpretation appropriate?

Not applicable

Are all the source data underlying the results available to ensure full reproducibility?

Yes

Is the study design appropriate and is the work technically sound?

Yes

Are the conclusions drawn adequately supported by the results?

Partly

Are sufficient details of methods and analysis provided to allow replication by others?

Yes

Reviewer Expertise:

Biotechnology.mmunotherapeutic discovery, engineering and development.

I confirm that I have read this submission and believe that I have an appropriate level of expertise to confirm that it is of an acceptable scientific standard, however I have significant reservations, as outlined above.

# Reviewer report: Reviewer response for version 1

**DOI:** 10.5256/f1000research.176528.r366733

**Published:** 2025-03-03

**Authors:** Jianlong Lou

**Affiliations:** 1 University of California San Francisco, San Francisco, California, USA

"Recombinant expression of receptor binding domains of all eight subtypes of botulinum neurotoxin type A for generation of antitoxins with broad reactivity" is a well-written original research report with clear citation of some current literature in the BoNT countermeasure development field.

 The work has some academic merit and potential field application, it is accurately presented with sufficient details, so other researchers should be able to replicate the experiments if desired. The relative simple software generated statistical analysis and its interpretation seems to be appropriate. And the conclusions drawn for in vitro activity were adequately supported by the results, but the author should not expand or imply the potential application for in vivo anti-toxin countermeasure efficiency without solid animal test data. 

 In vitro binding data and in vivo biological function data are somewhat related but of different dimensions.  The authors also need to explain the size of HC showing in Figure 1. Why does it look more like 100KD instead of 50KD if the molecular marker on the left side is correct? Although the designed fragment with 426 AA should have the molecular size close to 50KD?  Furthermore, Some publications such as the ones added below that discusses VHH specific for BoNT/A-LC or clinical trial using antibody based counter measure for BoNT/A will help reader to grasp the status and current situation of antitoxin development better.

Is the work clearly and accurately presented and does it cite the current literature?

Yes

If applicable, is the statistical analysis and its interpretation appropriate?

Yes

Are all the source data underlying the results available to ensure full reproducibility?

Yes

Is the study design appropriate and is the work technically sound?

Yes

Are the conclusions drawn adequately supported by the results?

Yes

Are sufficient details of methods and analysis provided to allow replication by others?

Yes

Reviewer Expertise:

Human antibody and animal based antibody engineering for BoNT and other antigens.

I confirm that I have read this submission and believe that I have an appropriate level of expertise to confirm that it is of an acceptable scientific standard.

## References

[1] : A single-domain llama antibody potently inhibits the enzymatic activity of botulinum neurotoxin by binding to the non-catalytic alpha-exosite binding region. *J Mol Biol* .2010;397(4) : 10.1016/j.jmb.2010.01.070 1106-18 10.1016/j.jmb.2010.01.070 20138889 PMC2903050

[2] : Safety and pharmacokinetics of XOMA 3AB, a novel mixture of three monoclonal antibodies against botulinum toxin A. *Antimicrob Agents Chemother* .2014;58(9) : 10.1128/AAC.02830-14 5047-53 10.1128/AAC.02830-14 24913160 PMC4135817

# Reviewer report: Reviewer response for version 1

**DOI:** 10.5256/f1000research.176528.r366732

**Published:** 2025-02-28

**Authors:** Ilias B Esmagambetov

**Affiliations:** 1 Gamaleya Research Center for Epidemiology and Microbiology, Moscow, Russian Federation

In general, the article is interesting for the target audience. However, the article contains very little data. In my opinion, it is not entirely correct to draw conclusions based only on ELISA data. Characterization of the interaction between the VHH and HC domain of BoNT/A using SPR or BLI methods would be very useful. I would recommend that the authors add at least one alternative method for characterizing the interaction of VHH with HC domain of BoNT/A.

Is the work clearly and accurately presented and does it cite the current literature?

Yes

If applicable, is the statistical analysis and its interpretation appropriate?

Not applicable

Are all the source data underlying the results available to ensure full reproducibility?

Yes

Is the study design appropriate and is the work technically sound?

Yes

Are the conclusions drawn adequately supported by the results?

Yes

Are sufficient details of methods and analysis provided to allow replication by others?

Yes

Reviewer Expertise:

mAbs, VHH, recombinant proteins, Adenoviral vectors, rAAV, gene therapy.

I confirm that I have read this submission and believe that I have an appropriate level of expertise to confirm that it is of an acceptable scientific standard, however I have significant reservations, as outlined above.
